# Brain Transcriptome Sequencing of a Natural Model of Alzheimer's Disease

**DOI:** 10.3389/fnagi.2017.00064

**Published:** 2017-03-20

**Authors:** Francisco Altimiras, Barbara Uszczynska-Ratajczak, Francisco Camara, Anna Vlasova, Emilio Palumbo, Stephen Newhouse, Robert M. J. Deacon, Leandro A. E. Farias, Michael J. Hurley, David E. Loyola, Rodrigo A. Vásquez, Richard Dobson, Roderic Guigó, Patricia Cogram

**Affiliations:** ^1^Faculty of Engineering and Sciences, Universidad Adolfo IbañezSantiago, Chile; ^2^Telefonica Research and DevelopmentSantiago, Chile; ^3^Centre for Genomic Regulation, Barcelona Institute of Science and TechnologyBarcelona, Spain; ^4^Universitat Pompeu FabraBarcelona, Spain; ^5^Institute of Psychiatry, Psychology and Neuroscience, King's College LondonLondon, UK; ^6^Laboratory of Molecular Neuropsychiatry, Institute of Cognitive and Translational Neuroscience (INCyT), INECO Foundation, Favaloro University, National Scientific and Technical Research CouncilBuenos Aires, Argentina; ^7^GeN.DDI LtdLondon, UK; ^8^Division of Brain Sciences, Centre for Neuroinflammation and Neurodegeneration, Imperial CollegeLondon, UK; ^9^National Center for Genomics and BioinformaticsSantiago, Chile; ^10^Faculty of Sciences, Institute of Ecology and Biodiversity, Universidad de ChileSantiago, Chile

**Keywords:** Alzheimer's disease, *Octodon degus*, RNA-seq, gene expression, animal models

## Introduction

Alzheimer's disease (AD) is a slowly progressive disease characterized by impairment of memory and eventually by disturbances in reasoning, planning, language, and perception. Ageing is the greatest risk factor for its development but mutations in amyloid precursor protein (APP), apolipoprotein E (APOE), microtubule-associated protein tau (MAPT) among others, are also a major factor (Blasko et al., 2004). The symptoms of AD result from neurofibrillary tangles that are composed of aggregates of hyper-phosphorylated tau protein and an increase in the production of amyloid-beta (Aβ) protein in the brain that leads to deposits of senile plaques. As such, there is a worldwide effort to find an effective disease-modifying treatment that can reverse symptoms and/or delay onset of the disease. Transgenic mouse models exist that mimic a range of AD–related pathologies, although none of the models fully replicate all pathological features of the human disease (Birch et al., 2014). Drugs developed using these mouse models have failed in phase III clinical trials (Mangialasche et al., 2010; Braidy et al., 2012; Saraceno et al., 2013). These failures question not only our accurate understanding of the disease (Castellani and Perry, 2012) but also the validity of the animal models upon which the drug discovery efforts are rooted (Windisch, 2014; Nazem et al., 2015).

Animal models have contributed significantly to our understanding of the underlying mechanisms of AD. To date, however, these findings have not resulted in target validation in humans and successful translation to disease-modifying therapies. The *Octodon degus (O. degus)* is a model that naturally integrates multiple AD pathological hallmarks like tau fibrilary tangles and β-amyloid deposits (Inestrosa et al., 2005, 2015; Deacon et al., 2015). The Aβ peptide sequence in *O. degus* is 97.5% homologous to the human Aβ peptide sequence (Inestrosa et al., 2005). The species presents acetylcholine (AChE)-rich pyramidal neurons in their forebrain, which decline in numbers during the progression to an AD-like behavioral state, similar to that seen in AD patients (Ardiles et al., 2012). Affected *O. degus* also present the characteristic medical signs and symptoms surrounding AD like macular degeneration, diabetes and circadian rhythm dysfunction (Laurijssens et al., 2013). Behavioral experiments have shown that the *O. degus* can also present behavioral deficits and neural alterations in the frontal cortex and aggression similar to those seen in patients with AD (Tarragon et al., 2013). Most importantly, the *O. degus* shows a correlation of expression with human AD- related genes making this model a powerful tool to characterize the effects of novel treatments for AD and identify new therapeutic targets. Our findings advance the use of the *O. degus* as an effective tool for AD research.

## Materials and methods

### Animals

In this study *O. degus* (Rodentia: Octodontidae) were captured from a natural population in central Chile, 30 km west of Santiago (Rinconada de Maipú, RM). In this environment, degus typically breed once per year in late autumn (May-June), with conceptions in late winter to early spring (September-October). The animals were captured as juveniles during early austral summer (November), when degus are 2–3 months old, with mesh made traps with a Sherman-trap type mechanism. Juveniles corresponded to individuals weighing 70–130 g (females) or 70–140 g (males), and hence it is possible to differentiate them categorically from adults, which weight above 130 g for females, 140 g for males (Ebensperger and Hurtado, 2005; Correa et al., 2016).

They were housed in standard metal cages 50 × 40 × 35 cm with a layer of wood shaving as bedding and containing a small metallic box (25 × 15 × 10 cm with a single entrance), under natural photoperiod (12 h light/dark; starting 7 a.m.) and in an air-conditioned animal facility at the Faculty of Sciences, University of Chile (Santiago, Chile). They were fed with a commercial rodent diet (Prolab RMH 3000, Lab diet). Water and food were provided *ad libitum* during the entire experimental period. Behavioral assessment of a population (*N* = 84) of 3-years old *O. degus* was performed using the burrowing test in an earlier work (Deacon et al., 2015), to address behavioral dysfunctions in activities of daily living (ADL) (Deacon, 2006, 2014). The population was divided in terms of burrowing performance into two groups: poor-burrowers (PB) and good-burrowers (GB). Brain extract samples were obtained from 8 females (four per each group). Brain soluble Aβ_1−42_peptide was determined for these 8 animals by MALDI-TOF MS as described in earlier work (Deacon et al., 2015). Samples were classified according to burrowing performance and brain soluble Aβ_1−42_ levels. Samples from four PB animals, with an increased level of soluble brain Aβ_1−42_ (AD-like group), and three samples from GB animals (with soluble levels of Aβ_1−42_ used as control group) were used for further analysis. Brain tissues were frozen directly in RNAlater solution (Ambion) and stored at −80°C until use. All procedures of capture, transportation, maintenance and experimentation followed the recommendations of the ethics committee of the Faculty of Sciences of the University of Chile, and complied with Chilean regulations (SAG-Chilean Agriculture and Livestock Service) as well as recommendations by the Animal Behavior Society.

### Total RNA and mRNA isolation

For total RNA extraction, PureLinkTM RNA Mini kit (Ambion, Life Technologies) was used according to the manufacturer's instructions. For genomic DNA digestion, RNase-Free DNase I (Ambion) was used. Total RNA samples were quantified in a Nanodrop 2000 spectrophotometer and integrity was evaluated by agarose gels electrophoresis with a standard protocol. For mRNA isolation from total RNA samples, MicroPoly(A) Purist kit was used according to the manufacturer's instructions. For sizing, quantitation and quality control of mRNA Bioanalyzer system (Agilent) was used, discarding samples with an RIN < 7.

### RNA-sequencing

RNA-seq was performed at the National Center for Genomics and Bioinformatics (Santiago, Chile) including library preparation (using SOLiD Total RNA-seq kit), fragmentation and PCR enrichment of target mRNA according to strand-specific protocols. Two batches of paired-end (75 × 35 bp) library sequencing were done in a SOLiD 5,500 × l system (Applied Biosystems).

### Gene prediction

We combined both *ab initio* and evidence-based approaches by using the Program to Assemble Spliced Alignments (PASA) and Evidence Modeler (EVM) to *de novo* annotate protein coding genes in *O. degus* genome assembly (v1.0) (Haas, 2003). In total 25,621 transcript models were generated by PASA from an initial set of 1,767,640 sequences including (1) 26,240 *O. degus* mRNA transcripts and (2) 1,741,759 ESTs and mRNA sequences from four species of rodents: mouse, guinea pig, Chinese hamster and rat. Transcript sequences for all five species were derived from the GenBank NCBI databases. We also used >30 million UniRef90 protein clusters and 26,259 highly curated rodent-specific Swiss-Prot proteins that were split-mapped to the *O. degus* genome by using SPALN2 (Iwata and Gotoh, 2012) and EXONERATE (Slater and Birney, 2005) with mouse-specific parameters. The protein-coding gene predictions were obtained on the latest repeat-masked reference assembly of the *O. degus* genome by using the existing mammalian-specific parameter files of four *ab initio* gene prediction programs: Augustus, Geneid, SGP2, and SNA (Guigó et al., 1992; Guigo et al., 2003; Parra et al., 2003; Stanke et al., 2006a,b). For SGP2, TBLASTX (Altschul, 1997) alignments between human (hg38) and *O. degus* were additionally used to improve the accuracy of gene predictions (Table S1). We also used external evidence for Geneid including PASA-derived introns, while for Augustus we used both PASA-derived intron and exon hints. Performance of all programs with and without external evidence was evaluated for accuracy on an artificial scaffold made up of 238 concatenated *O. degus* transcripts taken from the NCBI reference annotation. The alignments (transcript and protein) and *ab initio* gene models were combined into consensus CDS models using Evidence Modeler (Haas et al., 2008). The initial gene set was filtered to remove reference gene models supported exclusively by SNAP *ab initio* predictions. The single-exon consensus gene models derived from predictions solely supported by Geneid, SGP2 or AUGUSTUS with length < 300 nucleotides and an EVM score < 5 were also excluded. The weights of each given source were chosen empirically and based on suggestions contained in the EVM documentation. The highest weights were given to transcript alignments, followed by protein alignments and finally *ab initio* predictions (Augustus, Geneid, SGP2, and SNAP, Table S2). The consensus gene models were loaded into the PASA database and passed through five rounds of annotation updates to add UTRs and alternative splicing variants (Table S3).

### Functional annotation

Predicted protein-coding genes in the *O. degus* genome were further functionally annotated using an in-house developed, automatic pipeline. For each protein sequence we assigned protein signatures, orthology groups, as well as annotated metabolic pathways and reactions using an orthology-based approach. In this analysis we used InterProScan v.5 (Zdobnov and Apweiler, 2001) to scan though all available InterPro databases, including PANTHER, Pfam, TIGRFAM, HAMAP, and SUPERFAMILY and to specify different protein coding signatures in predicted protein coding genes. The protein signatures (protein families, regions, domains, repeats and sites) were further employed to investigate the classification and to assign biological functions to predicted proteins. Blast2GO (Götz et al., 2008) analysis was used to identify GO terms for the predicted proteins, while KEGG Automatic Annotation Server (KAAS) (Moriya et al., 2007) was employed to compare protein sequences against KEGG (Kanehisa et al., 2012, 2014) orthology (KO). In this analysis, KASS applied its bi-directional best hit (BBH) method in homology search against a representative gene set from 33 different species, including *Mus musculus* and *Cricetulus griseus*. KO identifiers were then used to retrieve the KEGG relevant functional annotation, such as metabolic pathways and external database references. In addition, we assigned the NCBI gene names from the *O. degus* reference NCBI annotation (ref_OctDeg1.0) to the predicted genes. This was done by taking NCBI gene names from the corresponding, NCBI annotated proteins, showing full sequence similarity. The sequence similarity was measured by assigning SHA1 checksum to each protein in both proteomes, followed by comparing those sums.

### Mapping *O. degus* brain RNA-seq samples

The *O. degus* RNA Sequences were aligned to a reference transcriptome obtained from the masked primary genome assembly version 1.0 (WGS Project: AJSA01) and the EVM-based genome annotation. The transcriptome sequence was prepared using RSEM version 1.2.12 (Li and Dewey, 2011) and projected from base space to SOLiD color space using SHRiMP version 2.2.3 (Rumble et al., 2009). The latter program was also employed to align SOLiD 75 × 35 bp pair-end sequenced reads to the reference transcriptome according to the following non-default parameters: -h 80% -o 10 -p opp-in –no-half-paired.

### Mapping human brain RNA-seq samples

The RNA-seq data set consisting of 6 human brain samples (3 AD subjects and 3 controls, University of Kentucky brain bank) was derived from the National Center for Biotechnology Information Sequence Read Archive database (ncbi.nlm.nih.gov/sra, accession no. SRA060572). Next, pair-end reads were mapped to the human reference transcriptome using our in-house pipeline that combines STARv.2.4.0.1 (Dobin et al., 2013) and RSEM version 1.2.12 (Table S4). The reference transcriptome was obtained from human reference genome hg38 and GENCODE v21 (Harrow et al., 2012) as a reference annotation.

### Expression profiling of human and *O. degus* brain samples

The number of reads mapped to each gene was calculated using SAMtools (Li et al., 2009). The statistical significance of expression profile for each gene between two groups was determined using edgeR, an open source R/Bioconductor package (Robinson et al., 2010). In this study, to estimate the significance of gene expression difference between AD or AD-like (PB) subjects and human controls or *O. degus* (GB) controls, the absolute value of log2 Ratio (logFC ≥ 1), log read count per million reads (logCPM > 1) and FDR < 0.05 were used as a criterion. Raw gene reads counts were also normalized to RPKM values (reads per kilobase per million mapped reads) using the RPKM formula described by Mortazavi et al. (2008).

### GO enrichment analysis for differentially expressed genes

Functional enrichment analysis for *O. degus* was performed using Fisher's exact test implemented in R programming language. The GO enrichment analysis for human was also done using R. The GOstats package was used to detect the significantly enriched GO terms for DEGs, which were compared to the full GENCODE 21 gene set. For both species, we further analyzed top 20 GO terms ranked by *p*-value with *p* < 0.05.

## Results

### Gene prediction and functional annotation

With the aim to enrich the current version of *O. degus* genome annotation with the new data obtained in this study, we performed *de novo* protein coding gene annotation. Our Evidence-Modeler (EVM) based annotation of repeat-mask *O. degus* genome (v1.0) resulted in prediction of 31,739 protein-coding genes, corresponding to 36,866 predicted transcripts and 36,575 proteins. The comparison of EVM-based and NCBI reference annotation revealed that the number of protein coding genes increased by 1.52-fold from 20,779 (NCBI) to 31,739 (EVM-based). Similar 1.40-fold change was reported for protein coding transcripts (26,248 for NCBI and 36,866 for EVM-based annotation) (Table S3).

Each protein sequence was functionally annotated using the in house automated pipeline. Annotation features were assigned to a total of 35,618 (97%) proteins, 30,661 (97%) genes), of these 29,847 (82%) of the proteins were assigned some GO terms (Tables S5, S6, Figures S1, S2). This functional annotation, including GO terms and KEGG orthology groups, allowed us to perform enrichment analysis for genes of interest.

### The brain transcriptome of AD-like *O. degus*

**Figure 1:**
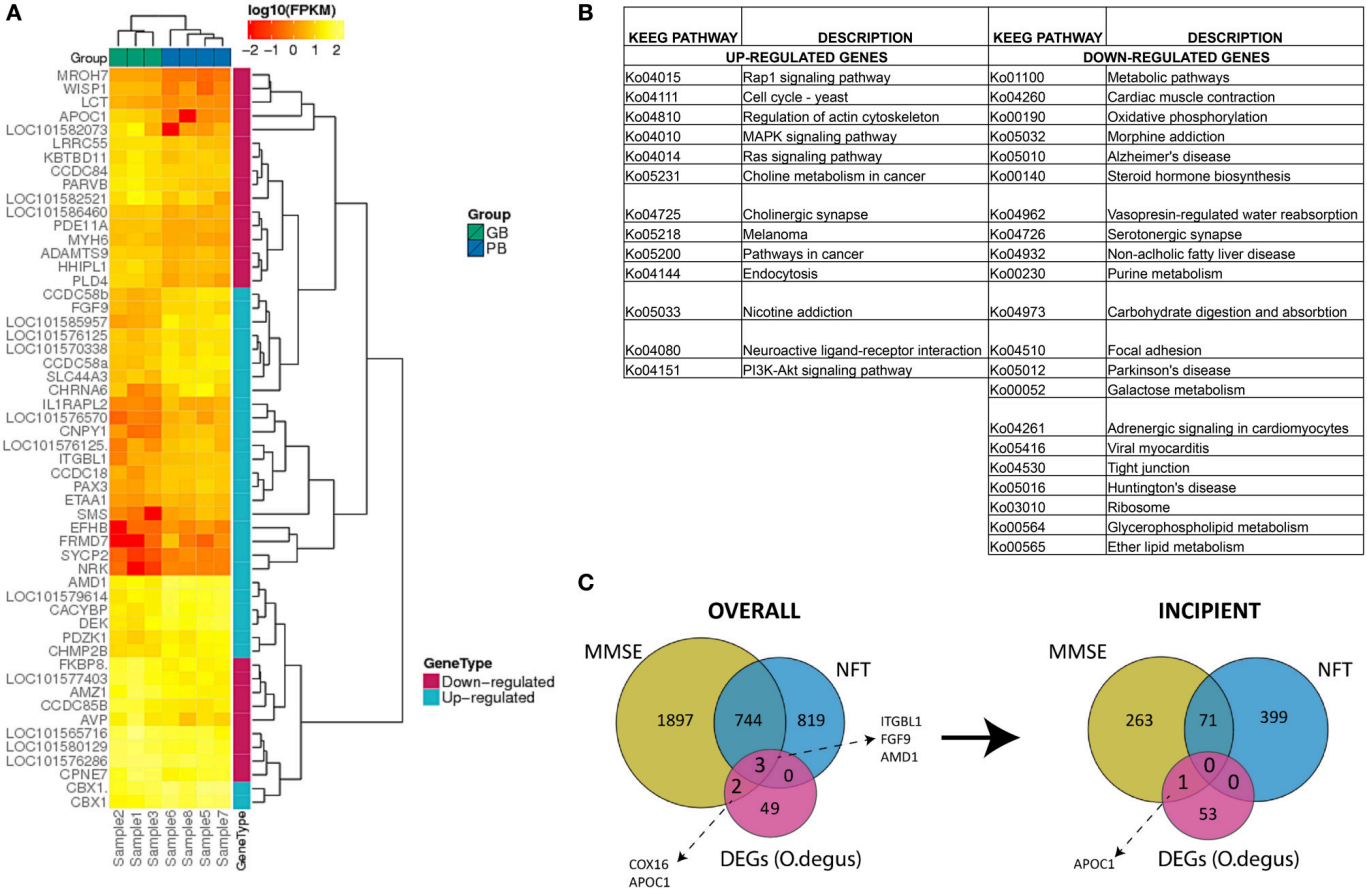
**(A)** Expression (FPKM values) analysis of 54 differentially expressed genes in PB group compared to GB group with two-way clustering applied. **(B)** Complete list of KEGG pathways corresponding to 29 up- and 25 down-regulated genes between PB and GB. Columns A and C: KEGG entry for up- and down-regulated genes, Columns B and D: Pathway names for up- and down-regulated genes. **(C)** Comparison of differentially expressed genes in PB vs. GB and AD-related human genes. Overall AD-correlated genes and AD-correlated genes with incipient AD stage.

After mapping the reads to the *O. degus* genome, we performed pairwise comparisons to measure the gene expression level differences between AD-like subjects and controls. As a result 54 DEGs were identified between those two groups. Statistical analysis revealed 29 genes to be up- and 25 to be down-regulated in our study (Table S10). The relative change in gene expression levels across AD-like and control samples is shown in Figure 1A.

To determine the function of DEGs, a Gene Ontology (GO) enrichment analysis was performed (Table S7). The up-regulated genes were enriched notably in chromosome related processes, which include chromosome condensation and chromosome organization. GO results showed also that up-regulated genes were largely involved in amine biosynthesis, in particular biosynthesis of polyamines. In contrast, most down-regulated genes were mainly engaged in ion homeostasis related processes, such as sodium-independent organic anion transport, hyperosmotic salinity response or regulation of cellular pH reduction. To fully determine which pathways could be directly affected in AD, DEGs were analyzed using KEGG PATHWAY Database (Braidy et al., 2012). The up-regulated genes were observed to be mainly part of signaling pathways including MAPK, Rap1, Ras and neurotransmission (cholinergic synapse), while down-regulated genes were part of pathways related to AD, Parkinson's disease and Huntington's disease (Figure 1B). Specifically down-regulation of *COX8A* gene. The *COX8A* encodes subunit VIIIA of cytochrome c oxidase, a crucial element for the formation of complex IV, which is a part of electron transport chain (ETC) in mitochondria. This is consistent with previously published data, as AD has been already linked to low activity of brain cytochrome c oxidase (Alleyne et al., 2011; Readnower et al., 2011). A substantial number of other down-regulated genes were also found to participate in different metabolic pathways.

### Comparison of human and *O. degus* AD-like brain transcriptomes

To gain insights to how *O. degus* could be employed as a natural model for studying the pathogenesis of AD, a human AD brain transcriptome was analyzed using RNA-seq data derived from hippocampus tissue of AD subjects and controls (Bai et al., 2013). Analysis of DEGs resulted in identification of 1,572 up and 1,391 down-regulated genes, which were further compared to DEGs identified for *O. degus* (Table S8). This comparison revealed overlap between seven genes, including *FGF9, WISP1* and *CPNE7* that are known to be linked with AD. Both *FGF9* and *WISP1* were observed to show the opposite expression changes for human and *O. degus*, while the altered expression of *CPNE7* was consistent between both species. Other common DEGs included one up-regulated gene: *ITGBL1*, and two down-regulated genes: *C1orf95, LRRC55*, as well as *IL1RAPL2*, which was down-regulated in *O. degus* brain and up-regulated in human hippocampus. To further compare human and *O. degus* AD-like brain transcriptomes, GO enrichment was employed. GO analysis revealed that that up-regulated genes for human were mainly involved in system and tissue development, in particular circulatory system and vasculature development (Table S9). This is to some extend consistent with biological processes described for *O. degus* up-regulated genes, as majority of enriched terms were biosynthesis related. Down-regulated genes were significantly enriched in neurological processes including signal transduction and neurogenesis. Similar to *O. degus*, AD affects glutamate secretion and glutamate receptor signaling pathways in human brain. Interestingly, genes involved in regulation of ion transport was also affected, supporting the relationship between cellular stress signals and AD.

### Comparison of *O. degus* DEGs and human AD-related genes

To investigate the potential role of *O. degus* DEGs in human AD progression, we compared our results to a set of AD-related genes, generated using Affymetrix expression microarrays across different stages of human AD: incipient, moderate and severe, as well as control samples (Blalock et al., 2011). AD-related genes were defined as genes, whose expression was positively or negatively correlated with results of MiniMental Status Examination (MMSE) and neurofibrillary tangles (NFT) counts across three AD categories (Blalock et al., 2004). Interestingly, five *O. degus* DEGs could be linked to AD-related genes in all subjects (overall correlation) this included *COX16, APOC1, ITGBL1, FGF9*, and *AMD1*. All five DEGs correlated with MMSE scores, while three: *ITGBL1, FGF9*, and *AMD1* were both MMSE and NFT-correlated. However, only *AMD1* showed a similar correlation with incipient AD (Figure 1C).

## Discussion

The purpose of the present study was to characterize the brain transcriptome of the *O. degus* to support its use as a natural model of AD, by comparing the transcriptomes of AD-like and healthy *O. degus*. This study is, to the best of our knowledge, the first report of *O. degus* brain whole transcriptome sequencing (RNA-seq). Our results revealed in *O. degus* a number of genes previously implicated in AD and related disorders indicating that this model is a suitable alternative to transgenic mice models for AD research. Because the AD-pathology can vary widely with animal age we carefully matched control and AD-like groups. Animals aged 3 years have optimal AD-like pathology development namely, the presence of beta-amyloid deposits as has been previously reported (Inestrosa et al., 2005; Ardiles et al., 2012; Deacon et al., 2015). This allowed for an acceptable signal dynamic for the experimental analysis. The sample size used for this study was calculated on the magnitude of the biological effect and the inherent variability of the target being measured, in this case the presence or absence of beta-amyloid deposits. We also considered the variability in the behavioral measures, which as previously reported (Deacon et al., 2015) showed clustering in two cohort of animals, AD-like and controls in the tests of daily living.

This work has identified up-regulated genes involved in AD including the CHRNA6 gene, which encodes a sub-type of neuronal nicotinic acetylcholine receptor widely associated to AD, which are mostly linked to Aβ aggregation theory and that was found previously affected in AD patients (Lombardo and Maskos, 2015). Another DEG AMD1 (which encodes an intermediate enzyme relevant to the polyamine biosynthesis) has an altered activity in AD and has also been implicated in schizophrenia and mood disorders, and was found up-regulated in affected AD-like *O. degus* (Morrison et al., 1993; Fiori and Turecki, 2008). The down-regulated genes found in the brains of AD-like *O. degus* included WNT1 inducible signaling pathway protein 1 (WISP1) (Varela-Nallar and Inestrosa, 2013). This gene previously implicated in neurodegeneration by regulation of mitochondrial signaling and apoptosis is a downstream target in the Wnt1 signaling pathway (Wang et al., 2012). Wnt signaling that involves Wnt1 and WISP1 is becoming recognized as a vital neuroprotective component during AD. WISP1 stops p53 mediated DNA damage and apoptosis offering neuronal protection by blocking cytochrome c release (Su et al., 2008; Wang et al., 2012). Mitochondrial-related genes were also found to be differentially expressed in AD-like *O. degus* brains, including the Cytochrome C Oxidase Subunit 8A (COX8A) that has been associated with the reduction of neuronal energy metabolism and mitochondrial dysfunction observed in AD patients by the inhibition of COX activity as a result of binding to Aβ-subunits (Lustbader et al., 2004; Liang et al., 2008).

The comparison of human and *O. degus* AD-like brain transcriptomes, revealed seven common genes that were deregulated. This included fibroblast growth factor 9 (*FGF9*), *WISP1* and Copine VII (*CPNE7*). Interestingly, the *FGF9* gene is known to be related to the MAPK pathway affected in AD patients (Antonell et al., 2013), and is one of the therapeutic targets currently used in AD treatment (Zhang et al., 2014).

Retinal abnormalities have also been reported to be associated to AD (Berisha et al., 2007). *O. degus* naturally develops cataracts, an eye dysfunction linked to neurodegeneration (Inestrosa et al., 2005, 2015; Braidy et al., 2015; Szabadfi et al., 2015). Our study revealed deregulated expression of two genes: *FRMD7* and *ADAMTS9*, which were previously associated to retinal abnormalities. The *FRMD7* gene plays role in retinal and neurite development, as well as in the neuronal outgrowth. It was also connected to X-linked infantile nystagmus, a disease characterized by abnormal eye function (Betts-Henderson et al., 2010). The *ADAMTS9* gene is also known to be involved in abnormal eye development. Extracellular matrix is a key mediator in the pathogenesis of age related macular degeneration and includes *ADAMTS9*. *ADAMTS9* activates *AKT* promoting photoreceptor degeneration and consequently membrane thickening and damage of retinal pigmentary cells. Moreover regulation of mTOR by ADAMTS9 leads to angiogenesis via VEGF A and TSP-1 this way regulating angiogenesis in the eye (Wight, 2005; Parry et al., 2009). Moreover, the activity disorders of ADAMTS9 regulation of the AKT/mTOR pathway leads to glycolysis and glucose uptake by increasing hypoxia-inducible factor (HIF)-1α (HIF1A) also been linked to AD (Avramovich-Tirosh et al., 2010). In humans, an ADAMTS9 gene variant is associated with type 2 diabetes (T2DM) is most frequently linked with aging, cognitive impairment, AD-associated neuronal APP-Aβ deposits (de la Monte and Wands, 2005). In relation to these results, the *O. degus* has insulin resistance and naturally develops type 2 diabetes and associated cataracts when fed with a diet high in glucose (Datiles and Fukui, 1989).

We have also identified *APOC-I* (apolipoprotein C-I) to be deregulated in AD-like *O. degus* brain samples. Notably, *APOC-I* is part of the APOE/C-I/C-IV/C-II gene cluster on the chromosome 19 (Smit et al., 1988) and APOE modulates its expression. This gene encodes a small apolipoprotein that is associated with Aβ plaques (Kamino et al., 1996). APOC-I plays a critical role in CNS homeostasis, since altered expression impairs memory (Abildayeva et al., 2008). Moreover, is to be highlighted that APOC-I co-localizes with Aβ plaques in the brain in AD (Abildayeva et al., 2008).

The pathogenic mechanism resulting in the onset and progression of neurodegenerative diseases like AD is often associated with genetic variants and mutations and interactions with environmental impacts, lifestyle risk factors, and slowly evolving molecular changes due to aging (Jicha and Carr, 2010; Duchen, 2012). Thus, a number of AD research avenues could be investigated in this model other than RNA analysis. For example post-translationally through modification of protein residues like phosphorylation.

In this study we focused in whole brain, so studies of additional brain regions are needed and may point to different genes and additional pathways. The RNA-seq uncovered multiple DE transcripts, several of these are novel and some have been previously implicated in AD like WISP1 and APOC1. Being the present work a pilot study we have probably uncovered only a fraction of what these data may reveal about this model and its link with AD supporting further work with this species. Future work is underway in our laboratory to elucidate the characterization of global gene expression of young and old *O. degus* by analysing tissue from different brain areas. To our knowledge this is the first *O. degus* brain transcriptome study. Salazar et al. (2016), compare the sequences of genes in relation to AD using published data from *O. degus*, human and other rodents. They analyzed tau, APP, Apolipoprotein E (APOE), Presenilin 1 (Psen1), and Aβ-peptide sequences in correlation with specific human variants associated with AD. Their findings revealed that *O. degus* have the arginine substitution present in the ApoE4 pathogenic human allele; Psen1 gene showed a greater relatedness between the isoforms of human, degus, guinea pig, and mole rat, compared to those of others rodents such the rat, and also described that *O. degus* Aβ-peptide sequence presents high homology with the human protein, differing in only one amino acid (Salazar et al., 2016). Furthermore, Deacon et al. (2015) shows that AD-like *O. degus* have high levels of Aβ-peptide aggregates, APP, TNF-a, IL-6, IFN-a, and the oxidative stress marker NFE2L2 when compared to healthy control *O. degus*, suggesting that AD-like *O. degus* present increased inflammation as is observed in human AD patients (Deacon et al., 2015). Overall the study of others and the present explorative transcriptome analysis revealed that the AD-like *O. degus* shares common affected genes with those have been implicated in human AD and therefore provides further characterization of the *O. degus* as a relevant natural model. *O. degus* shows a correlation of expression with human AD-related genes making this model a powerful preclinical tool to characterize target effects of novel therapeutics in AD. This study provides strong evidence to support the *O. degus* as a valuable natural model for preclinical work in AD research.

## Ethics statement

This study was carried out under the approval of the ethics committee of the Faculty of Sciences, Universidad de Chile, directed by Dr. Marco Méndez and integrated by:-Dr. Eduardo Friedman-Dr. Victoria Guixé-Dr. Madeleine Lamborot-Dr. Roberto Morales-Dr. Aurelio San Martín-Dr. Cecilia Vergara.

## Data availability

All the *Octodon degus* transcriptome data is available through the NCBI using accession PRJNA326273.

## Author contributions

FA and BU contributed to the experimental procedures, data analysis and the manuscript preparation. FC, AV, EP, SN, and DL contributed to created the bioinformatic pipeline for sequencing data analysis. LF contributed to the experimental procedures and data analysis. RMJD, MH, and RD contributed to comprehensive analysis of the results and to the manuscript preparation. RV, RG, and PC contributed to the funding of the project, the experimental design and the manuscript preparation.

### Conflict of interest statement

The authors declare that the research was conducted in the absence of any commercial or financial relationships that could be construed as a potential conflict of interest.
