# Supplementary material for: Brain Transcriptome Sequencing of a Natural Model of Alzheimer's Disease
Source: Front Aging Neurosci. 2017 Mar 20;9:64. doi: 10.3389/fnagi.2017.00064 (PMC5357652; doi:10.3389/fnagi.2017.00064)
Supplement: Supplementary file 1 [file DataSheet1.doc]

**Supplementary material**

**Brain transcriptome sequencing of a natural model of Alzheimer’s disease**

Francisco Altimiras^1,2,*,♯^, Barbara Uszczynska-Ratajczak^3,4,*^, Francisco Camara^3,4^, Anna Vlasova^3,4^, Emilio Palumbo^3,4^, Stephen Newhouse^5^, Robert M. J. Deacon^6,7^, Leandro A. E. Farias^1^, Michael J. Hurley^8^, David E. Loyola^9^, Rodrigo A. Vásquez^10^, Richard Dobson^5^, Roderic Guigó^3,4,♯^, and Patricia Cogram^6,7,♯^.

^1^Facultad de Ingeniería y Ciencias, Universidad Adolfo Ibañez, Santiago, Chile

^2^Telefonica Research and Development, Santiago, Chile

^3^Centre for Genomic Regulation, Barcelona Institute of Science and Technology, Barcelona, Spain

^4^Universitat Pompeu Fabra, Barcelona, Spain

^5^ Institute of Psychiatry, Psychology & Neuroscience, King's College London, London, UK

^6^ Laboratory of Molecular Neuropsychiatry, Institute of Cognitive and Translational Neuroscience (INCyT), INECO Foundation, Favaloro University, National Scientific and Technical Research Council (CONICET), Buenos Aires, Argentina

^7^ GeN.DDI Ltd, London, UK

^8^ Division of Brain Sciences, Centre for Neuroinflammation and Neurodegeneration, Imperial College, London, UK

^9^ National Center for Genomics and Bioinformatics, Santiago, Chile

^10^ Faculty of Sciences, Institute of Ecology and Biodiversity, Universidad de Chile, Santiago, Chile

*Shared first authorship

^♯^Corresponding authors

# **Supplementary tables**

**Table S1. Accuracy of gene prediction.** Accuracy of gene prediction on an *O. degus* “artificial scaffold” consisting of 238 concatenated *O. degus* test sequences (with 800 nucleotides of sequence between each of the gene models) using the ab initio programs geneid, AUGUSTUS and SNAP with their pre-existing Mammalian/H. sapiens parameter files (i.e. “mam/hs”). The accuracy of SGP2 (homology evidence-based prediction tool that used the genome of H. sapiens as reference) and that of Augustus (using RNASeq and transcript evidence i.e. “AUGUSTUS+hints”) were also tested for accuracy on the same set of sequences. Geneid (geneid+introns) and SGP2 (SGP2+introns) using introns as external evidence were also evaluated. (SN & SP: sensitivity & specificity at nucleotide level; SNe & SPe: sensitivity & specificity at exon level; SNg & SPg: sensitivity & specificity at gene level).

| Program**/param** | **SN** | **SP** | **SNe** | **SPe** | **SNg** | **SPg** |
| --- | --- | --- | --- | --- | --- | --- |
| Geneid **man/hs** | 0.83 | 0.75 | 0.65 | 0.69 | 0.09 | 0.06 |
| Geneid+intron **mam**/**hs** | 0.92 | 0.82 | 0.83 | 0.79 | 0.24 | 0.17 |
| SGP2 **odegus / Hs** | 0.90 | 0.82 | 0.77 | 0.73 | 0.12 | 0.08 |
| SGP2+intron **odegus / mam/hs** | 0.95 | 0.86 | 0.86 | 0.79 | 0.26 | 0.17 |
| Augustus+hints **mam/hs** | 0.87 | 0.94 | 0.81 | 0.90 | 0.33 | 0.35 |
| Augustus **mam/hs** | 0.81 | 0.84 | 0.68 | 0.75 | 0.06 | 0.07 |
| SNAP **mam/hs** | 0.83 | 0.45 | 0.57 | 0.32 | 0.03 | 0.01 |

**Table S2.** **Weights used by EVM to create a consensus CDS model *O. degus*.** The shortcuts interpretation: SPLAN2 uniprot90: SPLAN2 search against Uniprot90 proteins; SPALN2 uniprot-swissprot: SPALN2 against rodent uniprot/swissprot curated proteins; Exonerate uniprot-swissprot: exonerate against against rodent uniprot/swissprot curated proteins;

| **Type** | **Source** | **Weight** |
| --- | --- | --- |
| **ABINITIO_PREDICTION** | Augustus | 1 |
| **ABINITIO_PREDICTION** | AugustusHints | 1.75 |
| **ABINITIO_PREDICTION** | geneid | 1 |
| **ABINITIO_PREDICTION** | SGP2 | 1.25 |
| **ABINITIO_PREDICTION** | geneid+introns | 1.5 |
| **ABINITIO_PREDICTION** | SGP2+introns | 1.75 |
| **ABINITIO_PREDICTION** | SNAP | 0.3 |
| **PROTEIN** | SPALN2 uniprot90 | 5 |
| **PROTEIN** | SPALN2 uniprot-swissprot | 4 |
| **PROTEIN** | exonerate uniprot-swissprot | 4 |
| **TRANSCRIPT** | PASA | 10 |

**Table S3. Comparison between EVM-based and GNOMON-based protein coding gene annotation.** Statistics for two protein-coding annotations for *O. degus.*

| Annotation versions | ***O.degus 2a (EVM-generated)*** | ***O.degus (ncbi GNOMON) -protein-coding only-*** |
| --- | --- | --- |
| Genome length (Mbases) | **2,995.89** | |
| number of scaffolds | **7,134** | |
| Number of protein-coding genes | **31,739** | **20,779** |
| Gene density (genes/Kbase) | **0.0106** | **0.007** |
| Number of protein-coding transcripts | **36,866** | **26,248** |
| Transcripts/gene (range) (% genes with more than 1 transcript) | **1.16 (SD 0.72) (1 – 32) (9.24%)** | **1.26 (SD 0.94) (1 – 31)(15%)** |
| Number of transcripts with UTRs | **10,648** | **-** |
| Number of proteins | **36,575** | **26,248** |
| Number of complete proteins (%) | **33,858 (92.57%)** | **-** |
| Number/(%) proteins with similarity to sequences in the NCBI NR database (E=10-2; min. identity=25%) | **35,475 (97%)** | **-** |
| Avg. length of proteins (range) | **461.96 aa. (SD 593.73) (25 – 34,458)** | **577.56 aa. (SD 641.03) (23 – 34,357)** |
| Avg. length of full-length proteins (range) | **478.57 aa. (SD 602.27) (25 – 34,458)** | **-** |
| Number of partial proteins (not starting with "M") | **1842 (5.04%)** | **259 (0.98%)** |
| Avg. length of partial proteins (not starting with "M") | **253.11 aa. (SD 431.8)** | **-** |
| Number of partial proteins (no terminal STOP codon) | **1589 (4.34%)** | **(can’t determine as gnomon protein set has no clear STOP signal)** |
| Avg. length of partial proteins (no terminal STOP codon) | **213.91 aa. (SD 350.85)** | **-** |
| Number of partial proteins (not starting with an M -and- no terminal STOP codon) | **714 (1.95%)** | **-** |
| Avg. length of partial proteins (not starting with an M -and- no terminal STOP codon) | **158.83 aa. (SD 261.08)** | **-** |
| Number of partial proteins (not starting with an M -or- no terminal STOP codon) | **2,717 (7.43%)** | **-** |
| Avg. length of partial proteins (not starting with an M -or- no terminal STOP codon) | **254.96 aa. (SD 423.14)** | **-** |
| Number of protein-coding exons | **288,884** | **268,660** |
| Number of introns | **252,018** | **242,412** |
| Number of UTRs (spliced) | **19,003** | **-** |
| Number of single-exon genes | **10,114** | **3,156** |
| Number of multi-exonic transcripts (genes) | **26,752 (21,740)** | **23,092 (17,623)** |
| Exons/transcript (range) (excludes single-exon genes) | **10.42 (SD 10.50) (2 – 313)** | **11.49 (SD 10.35) (2 – 313)** |
| Introns/transcript  (range) | **9.42 (SD 10.50) (1 – 312)** | **10.49 (SD 10.35) (1 – 312)** |
| “spliced” UTRs/transcript (range) | **1.785 (SD 0.74) (1 - 5)** | **-** |
| Avg. length of introns (range) | **5,998 (SD 19,994.1) (21 – 734,060)** | **5,613.03 (SD 19,909.6) (30 – 1,116,408)** |
| Avg. length of mono-exonic genes | **519.27 (SD 430.56)** | **872.88 (SD 618.70)** |
| Avg. length of exons (excludes mono-exonic genes) | **165.37 (SD 233.34)** | **161.25 (SD 230.37)** |
| Avg. length of first exons | **230.78 (SD 336.07)** | **-** |
| Avg. length of internal exons | **149.24 (SD 194.59)** | **-** |
| Avg. length of terminal exons | **235.72 (SD 352.41)** | **-** |
| Avg. length of CDS (range) | **1,392.9 (SD 1,782.42) (75  – 103,074)** | **1,736.06 (SD 1,923.46) (69 – 103,074)** |
| Avg. length of UTRs (range) | **653.40 (SD 942.07) (1 - 11,857)** | **-** |
| Avg. length of primary transcripts | **43,714.8 (SD 107,530)** | **56,055.1 (SD 117,349)** |
| G+C content exonic (mono-exonic genes) | **49.72% (SD 7.63%)** | **51.85% (SD 8.56%)** |
| G+C content exonic (excludes mono-exonic genes) | **52.53% (SD 7.47%)** | **53.52% (SD 7.42%)** |
| G+C content exonic (first exons) | **53.62% (SD 10.85%)** | **-** |
| G+C content exonic (internal exons) | **51.27% (SD 9.67%)** | **-** |
| G+C content exonic (terminal exons) | **53.59% (SD 10.84%)** | **-** |
| G+C content intronic | **45.05% (SD 11.54%)** | **45.45% (SD 11.61%)** |
| G+C content genomic | **40.16% (SD 5.63%)** | |
| G+C content UTRs | **53,76% (SD 5%)** | **-** |

**Table S4. Non-default parameters for RNA-seq mappings. Non-default parameters used during mapping step of pair-end reads of human brain AD subjects and control samples with STAR 2.4.0.1. First column refers to the name of the parameter, while the second to its value.**

| **Parameter** | **Value** |
| --- | --- |
| outSAMunmapped | Within |
| outFilterType | BySJout |
| outFilterMultimapNmax | 20 |
| outFilterMismatchNmax | 999 |
| outFilterMismatchNoverReadLmax | 0.04 |
| alignIntronMin | 20 |
| alignIntronMax | 1000000 |
| alignSJDBoverhangMin | 1 |
| readFilesCommand | zcat |

**Supplementary Table S5. Functional annotation statistics.** Abbreviature KO – KEGG orthology groups.

| Number of proteins/genes | 36,575 / 31,739 |
| --- | --- |
| **Annotated proteins/genes** | **34,571 (94.5%) / 30,336 (95.5%)** |
| Proteins with Interpro signatures | 33,800 (92.4%) |
| Proteins with Blast2GO or KEGG definition | 23,936 (65.4%) |
| Proteins with Blast2GO definition | 16,737 (45.7%) |
| Proteins with KEGG definition | 14,756 (40.3%) |
| Proteins assigned to KO groups | 14,879 (40.6%) |
| Proteins with GO terms association | 28,988 (79.2%) |
| Conserved domains signatures | 31,874 (87.1%) |
| Conserved features signatures | 15,017 (41%) |

**Supplementary Table S6. GO term annotation.**  **A**. Number of GO terms associated to each ontology **B**. Top 10 GO terms more frequently associated to proteins grouped by GO term type.

**A.**

| **Term type** | **Number of proteins** |
| --- | --- |
| Biological process | 22,200 |
| Cellular component | 20,110 |
| Molecular function | 27,220 |
| **All** | **29,847** |

**B**

| **GO term id** | **GO term description** | **# Proteins** |
| --- | --- | --- |
|  | **Biological process** |  |
| GO:0006412 | translation | 2938 |
| GO:0006355 | regulation of transcription, DNA-templated | 1865 |
| GO:0007186 | G-protein coupled receptor signaling pathway | 1505 |
| GO:0006414 | translational elongation | 1073 |
| GO:0055114 | oxidation-reduction process | 1016 |
| GO:0006413 | translational initiation | 904 |
| GO:0007165 | signal transduction | 902 |
| GO:0006468 | protein phosphorylation | 882 |
| GO:0000184 | nuclear-transcribed mRNA catabolic process, nonsense-mediated decay | 830 |
| GO:0006614 | SRP-dependent cotranslational protein targeting to membrane | 823 |
|  | **Molecular function** |  |
| GO:0005515 | protein binding | 6905 |
| GO:0003735 | structural constituent of ribosome | 3023 |
| GO:0005524 | ATP binding | 2431 |
| GO:0003676 | nucleic acid binding | 2166 |
| GO:0008270 | zinc ion binding | 1982 |
| GO:0000166 | nucleotide binding | 1785 |
| GO:0003677 | DNA binding | 1755 |
| GO:0046872 | metal ion binding | 1706 |
| GO:0004930 | G-protein coupled receptor activity | 1311 |
| GO:0003723 | RNA binding | 1280 |
|  | **Cellular component** |  |
| GO:0005622 | intracellular | 3658 |
| GO:0005634 | nucleus | 3517 |
| GO:0016021 | integral component of membrane | 3502 |
| GO:0005840 | ribosome | 2927 |
| GO:0005737 | cytoplasm | 2338 |
| GO:0016020 | membrane | 2165 |
| GO:0005829 | cytosol | 1641 |
| GO:0005886 | plasma membrane | 1487 |
| GO:0005730 | nucleolus | 1429 |
| GO:0022625 | cytosolic large ribosomal subunit | 1007 |

**Table S7. GO-terms enrichment for differentially expressed genes in *O. degus*.** Biological processes overrepresented by up- and down-regulated genes identified in *O. degus* brain samples. GO terms shown are those significantly overrepresented (pvalue < 0.05) by genes showing differential expression between AD-like subjects and controls. Categories are sorted by p-value.

**TABLE IN ADDITIONAL FILE 2**

**Table S8. GO terms enrichment for differentially expressed genes in human samples.** Biological processes overrepresented by up- and down-regulated genes identified in human brain samples. GO terms shown are those significantly overrepresented (pvalue < 0.05) by genes showing differential expression between AD subjects and controls. Categories are sorted by p-value.

**TABLE IN ADDITIONAL FILE 2**

**Table S9. Genes differentially expressed between human samples.** The complete list of 2963 human genes displaying differential expression between AD subjects and controls. Genes are ranked by FDR (FDR < 0.05).

**TABLE IN ADDITIONAL FILE 2**

**Table S10. Pairwise comparisons to measure the gene expression level differences between AD-like degus and controls.**

**TABLE IN ADDITIONAL FILE 2**

**Supplementary figures**


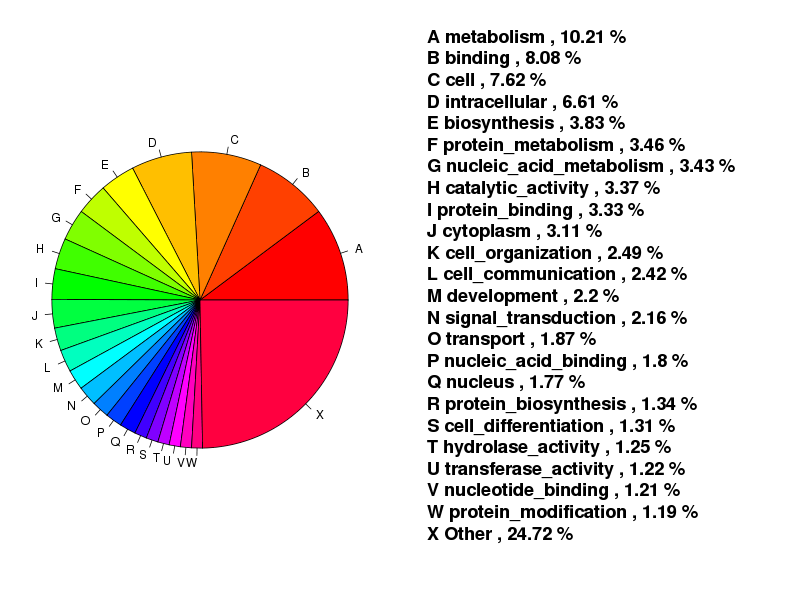


**Figure S1. Gene ontology mapping of O*. degus* genes.** The GO terms were mapped into the general GO slims without top level categories – biological process, molecular function, cellular component.


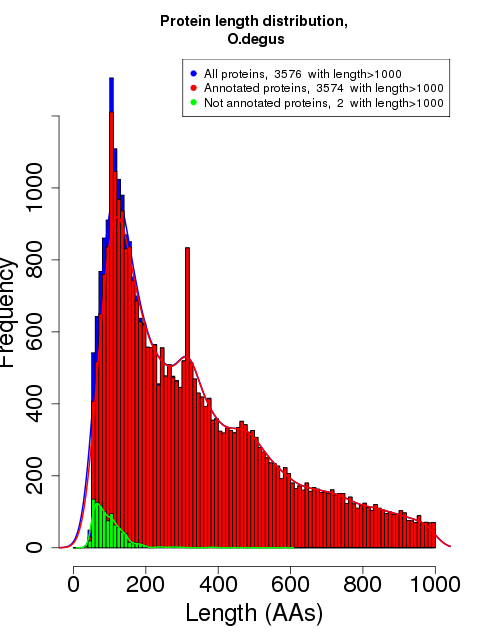


**Figure S2**. **Distribution of functionally annotated and non-annotated proteins.** Number of annotated and non-annotated sequences in relation to their length. The blue color correspond to all proteins, red – annotated proteins and green to the non-annotated proteins.
